# Predicting resource-dependent maternal health outcomes at a referral hospital in Zanzibar using patient trajectories and mathematical modeling

**DOI:** 10.1371/journal.pone.0212753

**Published:** 2019-03-05

**Authors:** Devika Nadkarni, Avijit Minocha, Harshit Harpaldas, Grace Kim, Anuraag Gopaluni, Sara Gravelyn, Sarem Rashid, Anna Helfrich, Katie Clifford, Tanneke Herklots, Tarek Meguid, Benoit Jacod, Darash Desai, Muhammad H. Zaman

**Affiliations:** 1 Department of Biomedical Engineering, Boston University, Boston, Massachusetts, United States of America; 2 Department of Obstetrics & Gynecology, University Medical Center Utrecht, Utrecht, the Netherlands; 3 O’Neil Institute for National and Global Health Law, Georgetown University, Washington DC, United States of America; 4 Department of Obstetrics, Amalia Children’s Hospital Radboudumc, Nijmegen, the Netherlands; 5 Howard Hughes Medical Institute, Boston University, Boston, Massachusetts, United States of America; Aga Khan University, KENYA

## Abstract

Poor intra-facility maternity care is a major contributor to maternal mortality in low- and middle-income countries. Close to 830 women die each day due to preventable maternal complications, partly due to the increasing number of women giving birth in health facilities that are not adequately resourced to manage growing patient populations. Barriers to adequate care during the ‘last mile’ of healthcare delivery are attributable to deficiencies at multiple levels: education, staff, medication, facilities, and delays in receiving care. Moreover, the scope and multi-scale interdependence of these factors make individual contributions of each challenging to analyze, particularly in settings where basic data registration is often lacking. To address this need, we have designed and implemented a novel systems-level and dynamic mathematical model that simulates the impact of hospital resource allocations on maternal mortality rates at Mnazi Mmoja Hospital (MMH), a referral hospital in Zanzibar, Tanzania. The purpose of this model is to provide a rigorous and flexible tool that enables hospital administrators and public health officials to quantitatively analyze the impact of resource constraints on patient outcomes within the maternity ward, and prioritize key areas for further human or capital investment. Currently, no such tool exists to assist administrators and policy makers with effective resource allocation and planning. This paper describes the structure and construct of the model, provides validation of the assumptions made with anonymized patient data and discusses the predictive capacity of our model. Application of the model to specific resource allocations, maternal treatment plans, and hospital loads at MMH indicates through quantitative results that medicine stocking schedules and staff allocations are key areas that can be addressed to reduce mortality by up to 5-fold. With data-driven evidence provided by the model, hospital staff, administration, and the local ministries of health can enact policy changes and implement targeted interventions to improve maternal health outcomes at MMH. While our model is able to determine specific gaps in resources and health care delivery specifically at MMH, the model should be viewed as an additional tool that may be used by other facilities seeking to analyze and improve maternal health outcomes in resource constrained environments.

## Introduction

Every day, close to 830 women die of preventable maternal complications. Nearly all of these maternal deaths occur in developing countries, the majority of which occur specifically in sub-Saharan Africa [1–3]. One in 16 women in these regions die in pregnancy or childbirth as a result of these complications– 175 times the maternal mortality risk of high income countries [4]. Poor intra-facility maternity care is becoming a major contributor to overall maternal mortality as an increasing number of women are persuaded to give birth at health facilities [5]. Barriers to adequate care during the ‘last mile’ of healthcare delivery are the result of deficiencies at multiple levels: education, staff, medication, facilities, and delays in receiving care. The contribution of each of these factors is difficult to analyze in settings where even basic data registration is lacking [6]. It is, however, crucial to understand how each factor contributes to a facility’s maternal health outcomes, as “more of everything” is not a viable strategy with limited financial resource availability,.

To assess the burden of poor maternal health, the WHO has created the Maternal Severity Index (MSI), which indicates the probability of death amongst all severe maternal outcomes, and the Workforce Indicators of Staffing Need (WISN), which calculates the number of new health workers needed to meet the demand [7, 8]. Both the MSI and WISN have been utilized in previous studies to measure the relationship of in-hospital maternal health complications and human resources to maternal mortality [7, 9]. In addition to human resources, delays in receiving monitoring and treatment for complications in pregnancy can lead to adverse maternal health outcomes [10, 11]. Studies have explored different delay models to identify causes of critical delays in delivery that result in adverse maternal health outcomes [12–15]. However, current research has not clearly identified possible solutions at the facility level to address these institutional delays. While preventable maternal mortality is linked to the availability of resources at maternal health facilities, the relationship has not been quantitatively examined [10]. To address this gap, we have developed a dynamic computational simulation of a maternity ward that can be used as a tool to quantitatively understand how preventable maternal mortality is linked to resource availability. This model can serve as a robust decision aid for hospital administrators and policymakers seeking to improve maternal mortality at the facility-level within a constrained set of resources. It incorporates key parameters of the health care facility such as medications, supplies, staff and physical infrastructure to understand how they may be optimized to minimize maternal mortality.

To demonstrate its utility for a real maternal health facility, we have developed the model to reflect the structure and processes of the maternity ward at Mnazi Mmoja Hospital (MMH) in Zanzibar. MMH is the only public referral hospital in Zanzibar, a semi-autonomous archipelago of Tanzania. It receives referrals from other health centers to treat high-risk patients, but many patients without complications also self-refer to the hospital. As such, it serves a large portion of Zanzibar’s entire 1.3 million people population. The maternity ward in MMH is burdened with approximately 12,000 deliveries per year and limited numbers of skilled health workers and supplies [16]. Previous studies have found the maternal mortality ratio at MMH to be 457 deaths per 100,000 live births—over twice the global mortality ratio of 216 deaths per 100,000 [16, 17]. While there is an understanding that delays in treatment are often due to lack of trained staff, there are no systems in place to account for the number of staff on shift, depleted medical supplies, and inconsistent documentation of patient records [16]. Therefore, it is difficult to predict how changing each of these factors in isolation or in tandem could impact maternal health outcomes.

Our mathematical model incorporates these factors to analyze resource use and maternal mortality for a population reflecting that found at MMH. The purpose of this model is to provide a tool that enables hospital administrators and public health officials to generate quantitative data on how patient outcomes are impacted by resource allocation and availability within the maternity ward to help them optimize resource use. This model will be able to determine specific gaps in resources and health care delivery, and this information may be used for targeted and specific interventions to improve maternal health outcomes. With such targeted interventions backed by evidence from the model, hospital staff, administration, and the Ministry of Health can work collaboratively to enact policy and operational changes to improve maternal health outcomes.

## Methods

De-identified patient data spanning approximately six months was collected from medical records at Mnazi Mmoja Hospital. This research was approved by ZAMREC, the research authority in Zanzibar, Tanzania on July 18 2017; Protocol No. ZAMREC/0001/JUN/17. A stepwise, iterative, object-oriented program was developed to simulate the workflow and patient treatment process at the maternity ward at MMH. In order to ensure the algorithm accurately reflects a patient’s stay at the hospital and the resources used in treatment, it was developed in close collaboration with doctors, nurses, and clinical researchers at MMH. The model allows users to define resource allocations, patient load and incoming morbidity distributions over various shifts, as well as specific treatment plans based on patient status. A full list of key variables, classifications, and measures used throughout the model are summarized in Table 1. These factors are tracked and updated over a simulated duration of time, providing users with information on short-term and long-term maternal mortality impacts.

**Table 1:** Description of key variables, clinical classification, and outcome measures used in the model.

Term	Description
*Key variables*	
Probability of Mortality	A value between 0 and 1 indicates the severity of the patient’s condition and represents how likely the patient’s complication is to becoming fatal over their entire stay at the hospital, where 0 represents a 0% chance of death and 1 represents a 100% chance of death.
Deterioration rate	A numerical value that is associated with each complication that indicates how quickly the complication will become fatal if left untreated.
Treatment plan	An object assigned by the model to each patient based on the probability of mortality of each of their complications. It describes the types of medications, medication dosages, the number of nurses and doctors required to execute the plan, how frequently each staff member needs to be with a patient, the total duration in time (in simulation cycles) until completion of the treatment plan, and a treatment efficacy that determines how rapidly a patient’s probability of mortality will improve when treated.
Treatment efficacy	An integer between 0 and 1 that is unique to each treatment plan and represents the extent to which the treatment plan decreases a patient’s probability of mortality. An efficacy of 1 is most efficacious resulting in a probability of mortality of 0, and an efficacy of 0 indicates that the treatment plan does not decrease the probability of mortality.
Severity distribution	A normal distribution unique to each complication that is used to assign a random severity for each patient that presents with a given complication upon admission.
Cycle	A single cycle is fifteen minutes of simulated time in the model.
*Clinical classification*	
Potentially-life threatening complication	A complication in pregnancy that could be fatal if untreated.
Severe maternal outcome	An outcome in which the patient comes closest to maternal death, and may survive (maternal near-miss) or may not (maternal death)
Maternal-near miss	A case in which a patient comes close to maternal death but does not die.
Maternal death	Maternal death is the death of a woman while pregnant or within 42 days of termination of pregnancy, irrespective of the duration and site of the pregnancy, from any cause related to or aggravated by the pregnancy or its management but not from accidental or incidental causes.
*Outcome measures*	
Mortality Rate	The percentage of fatal cases in the specified patient cohort
Case fatality rate	The percentage of fatal cases for a given complication
Complication Incidence Rate	The percentage of cases with a given complication in the entire patient cohort

In order to construct the model, patient records from the MMH maternity ward between July 2016 and September 2016 were anonymized and digitized to create a database of 343 patients with potentially life-threatening complications and 2,285 patients with uncomplicated deliveries. Potentially life-threatening complications included preeclampsia, eclampsia, antepartum hemorrhage, postpartum hemorrhage, rupture of the uterus, sepsis or systemic infection, cardiomyopathy, and anemia with a hemoglobin level below 7 g/dL. In addition to patient records, information on staffing levels over ten days at the hospital, staff scheduling, and ward inventory data detailing the stocks of all medications supplied to the ward was collected. Data on case fatality rates and causes of death were used from a previous clinical study at MMH’s maternity ward [16]. Complication incidence rates, average duration of patient stay for each condition, the rate of occurrence of multiple complications in a patient, and the number of maternal near-miss cases were calculated from this database of patient records.

To appropriately scope the model, only five complications—postpartum hemorrhage, preeclampsia, eclampsia, rupture of the uterus, and sepsis—were considered as discussed in the inclusion criteria set of the World Health Organization’s near-miss criteria [18–21]. The model algorithm was designed to replicate patient admission, treatment, and triage mechanisms in the maternity ward. Finally, the model was calibrated to reflect the current maternal health outcomes at MMH as determined by Herklots et al [16]. This was done by adjusting the distribution for the relative severity of each complication (severity distribution), how effectively treatment alleviated complications (treatment efficacy), and how quickly a patient’s condition deteriorated without treatment (deterioration rate).

### Model foundation and structure

The foundation of the model is an iterative simulation that accounts for the stochastic nature and nonlinear impacts of hospital admissions and condition severities, tracking both staff resources and medicines stocks in the process. Each iteration or cycle of the model represents fifteen minutes at the hospital maternity ward—the shortest duration necessary for a single intervention outlined in clinician treatment plans for each complication. Each cycle consists of four key steps:

1. Admission of a new patient
2. Allocation of resources to patients
3. Evaluation of patient health and resource redistribution to hospital
4. Restocking hospital resources

#### Admission of new patients

**Fig 1:**
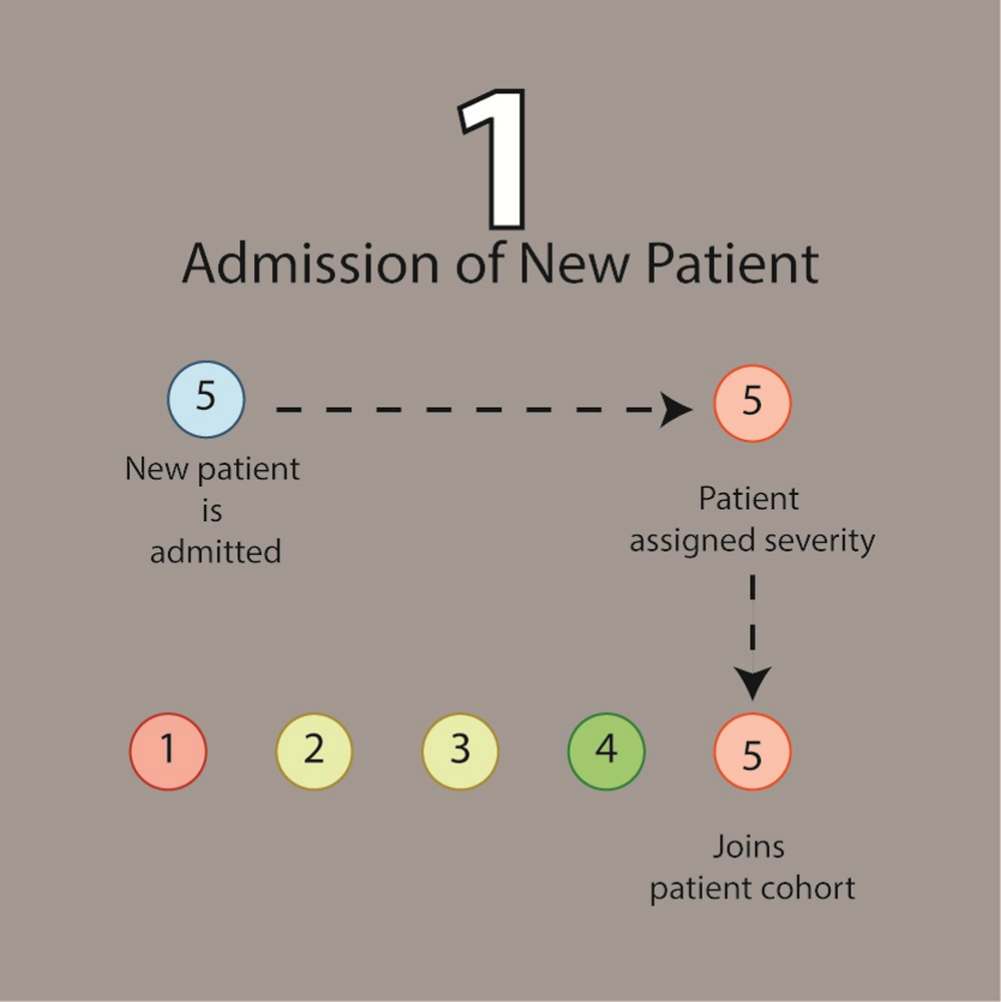
Schematic of the first step in the algorithm—the creation and admission of a new patient (blue) to the simulated ward with a high probability of mortality (red), a moderate probability of mortality (orange and yellow), or low probability of mortality (green).

Beginning each cycle, the model simulates the intake of new patients to the existing cohort based on a probability of admission that varies from morning, afternoon, to evening (Fig 1). These numbers were calculated as the probability of a patient arriving every 15 minutes throughout the day based on real patient admission data recorded from the maternity ward at MMH over ten days. If a patient is added to the cohort, the model randomly ascribes complications and associated severities to the incoming patient (if any) based on predefined incidence rates and severity distributions for each complication. Incidence rates were determined from the deidentified patient data, and corresponding severity distributions for complications at patient admission were formulated as follows: each complication’s severity distribution is modeled as a truncated normal distribution on the closed interval of (0, 1) and centered around a mean probability of mortality corresponding to actual case fatality rates found by Herklots et al [16]. The standard deviation for each distribution was defined based on the concept that a small proportion of incoming patients would arrive with a probability of mortality associated with a severe maternal outcome (SMO)–patients that either meet the near miss criteria or become maternal deaths. As a base assumption, this proportion was set to 2.5% (two standard deviations above the mean), and the probability of mortality at this point was fixed to the chance of dying if classified as an SMO patient; this was calculated based on a study by Herklots et al as the proportion of maternal deaths among all SMO patients [16].

Once a set of probability of mortalities for an incoming patient has been randomly selected from severity distributions for each of the patient’s complication, a composite probability of mortality for the patient, *p*_*c*_, is calculated under the simplification that each condition contributes independently to the patient’s health. For a patient exhibiting two simultaneous conditions with probabilities of mortality of p_1_ and p_2_, the probability of survival for each condition is (1-p_1_) and (1-p_2_), respectively. The total probability of survival is then (1-p_1_)(1-p_2_), making the total probability of mortality, *p*_*c*,_ 1 –(1-p_1_)(1-p_2_).This composite probability of mortality is then utilized in the next section of the model to prioritize resource allocations to each patient.

#### Allocation of resources to patients

**Fig 2:**
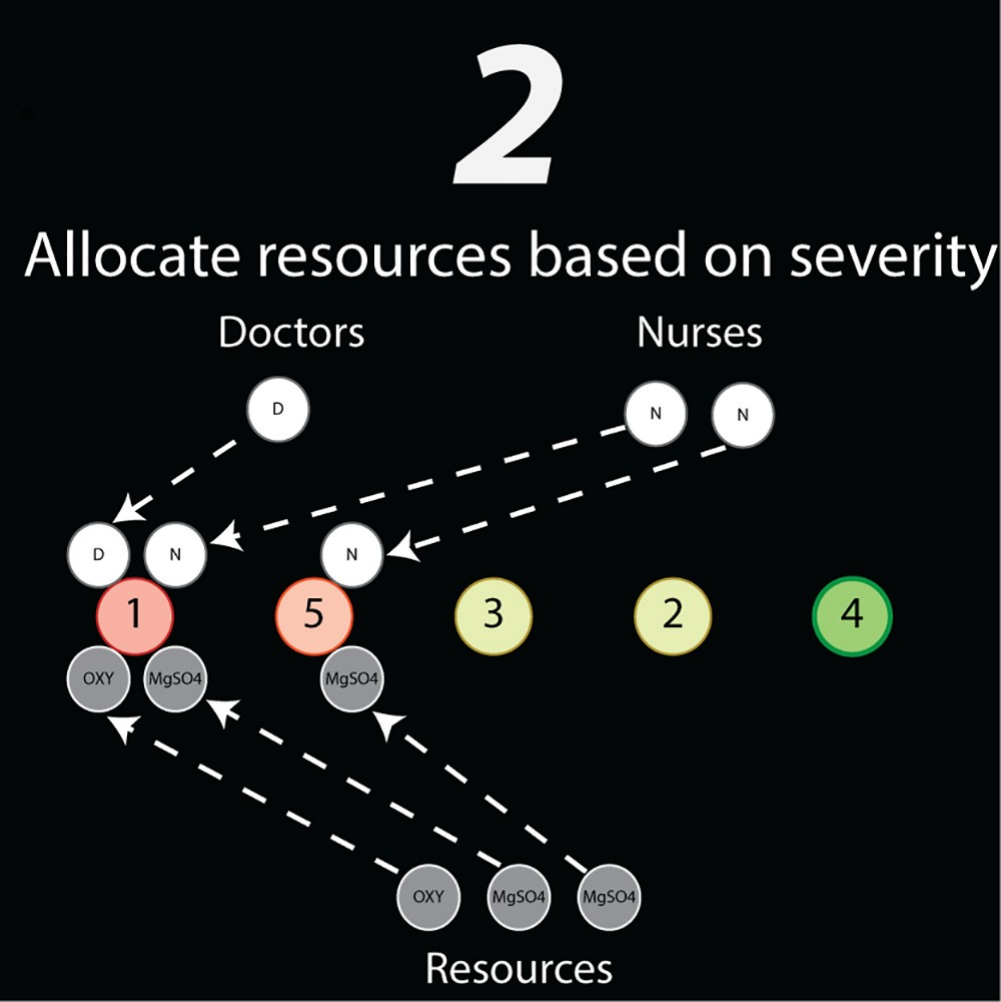
Schematic of the second step of the algorithm—allocation of resources to patients as prescribed by their respective treatment plans, starting with the patients with the highest probability of mortality (red) and ending with patients with the lowest probability of mortality (green).

Once new patients have been added to the cohort, the model determines the resources required by the cohort based on the assignment of treatment plans for each patient’s condition(s) (Fig 2). These treatment plans outline specific resource requirements such as frequencies and dosages of medicines, number and frequency of nurse visits, and number and frequency of doctor visits (S2 Table). They are specific to the patient’s current status and conditions and are in effect for a predefined duration of time or model cycles, but are reevaluated from cycle to cycle based on whether the patient improves, worsens, or successfully completes a plan. Treatment plans may also be linked, such that successful completion of one treatment plan moves the patient to a predefined subsequent treatment plan.

With a treatment plan and associated resources determined for each patient, the model begins treating patients in descending order of composite probability of mortality, with the patients with the highest probability of mortality being treated first; this is consistent with the standard protocol at MMH. Each patient is treated only if *all* medication and staff resources required by the treatment plan are available. If a single human or material resource is missing from the standard treatment plan, the patient will not be treated. Furthermore, doctors and nurses that are specified for consecutive treatment cycles in a particular treatment plan will stay with the patient and will not be returned to hospital staff resource pool until their visitation is complete.

As the patient progresses through treatment, the probability of mortality, *p*, for each of her conditions decreases with each cycle based on the efficacy of her treatment plan, η. For each cycle that she is treated, the patient’s probability of mortality is calculated as p = p_i_(1—η), where p_i_ is the probability of mortality at the start of the cycle and p is the new probability of mortality after treatment that cycle. Over successive cycles of treatment, this approach leads to a geometric decay of the probability of mortality with the form *p*(*c*) = *p*_i_^*rc*^, where *p*_i_ is the probability of mortality at *c* = 0, *c* is the number of successive cycles of treatment, and *r* is the rate constant and is related to the treatment efficacy by *r* = 1—η. The treatment efficacy for each treatment plan was calibrated by ensuring that with complete and timely treatment, a patient’s mean probability of mortality for each complication would decrease to zero by the end of a hospital stay (numerically chosen as 10^−10^).

In the case that enough resources are not available to carry out the treatment plan for a given cycle, treatment is not omitted that cycle, causing the probability of mortality for each complication to worsen according to $p\;=\;{p_{i}}^{\frac{\lambda }{\lambda +c}}$, where *p*_*i*_ is probability of mortality for the condition at the beginning of the cycle, *λ* is the deterioration rate, and *c* is the number of consecutive cycles that the patient has not received treatment. Over successive cycles without treatment, this leads to a sigmoidal increase in the probability of mortality on the open interval of (0,1), with the form $p(c)\;=\;{p_{i}}^{\frac{\lambda^{c}\Gamma (\lambda +1)}{\Gamma (\lambda +c+1)}}$, where *p*_i_ is the probability of mortality for the condition for the first cycle that treatment is not provided, *c* remains the number of successive cycles treatment is not provided and Γ is the mathematical gamma function. Deterioration rates for each condition were initially estimated qualitatively from patient records based on the mean length of time required for a complication to become fatal, after which they were adjusted to reflect expected mortality outcomes for each complication as observed in the patient files collected from MMH.

#### Evaluate patient health and redistribute resources

**Fig 3:**
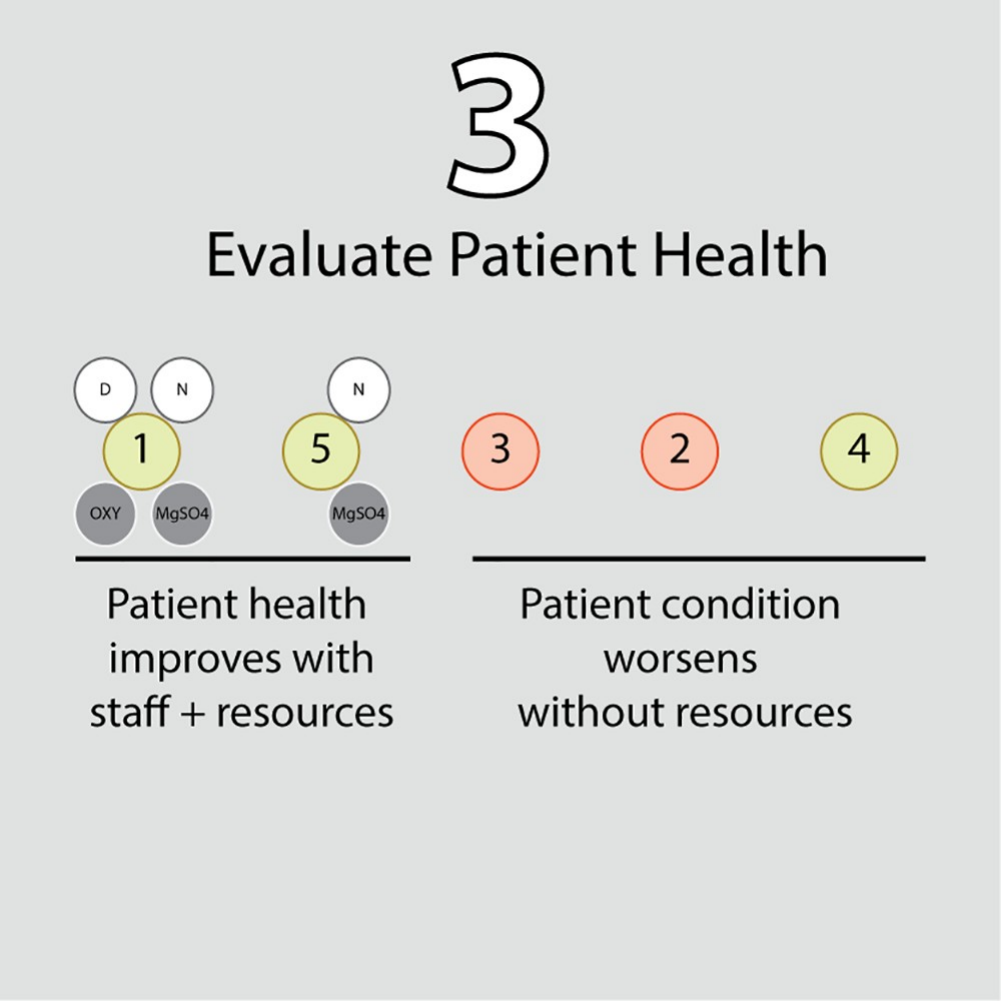
Schematic of the third step in the algorithm—the probabilities of mortality for patients who were treated will decrease as per the efficacy of their assigned treatment plans, while the probabilities of mortality for patients that did not get treated due to insufficient resources will increase based on the deterioration rates of their respective complications (if any).

In the third stage, the model iterates through the patient cohort and stochastically determines which patients survive based on the selection of a uniform random variate for each patient (Fig 3). If the random variate is greater than the patient’s probability of mortality, the patient survives. Because each patient’s composite probability of mortality, *p*_*c*_, is considered their chance of death over the full length of their remaining hospital stay, a scaled probability of mortality, *p*, is used to reflect their chance of death within only the current cycle. This scaled probability of mortality is calculated based on a binomial distribution, where the composite probability of mortality is the cumulative probability of dying at any cycle and is given by pc=∑k=1n(nk)pk(1-p)n-k, where *n* is the number of remaining cycles in the patient’s intended hospital stay. To solve for *p*, we may instead consider the probability of surviving, *p*_*s*_, where *p*_*c*_ = 1 − *p*_*s*_, and by the binomial distribution in the case of *k* = 0 over *n* cycles, *p*_*s*_ = (1 − *p*)^*n*^. Hence, we find that *p* is related to *p*_*c*_ by $p\;=\;1-\sqrt[{n}]{1-p_{c}})$. After assessing the survival of each patient based on this scaled probability of mortality, any staff resources that were associated with patients that did not survive are promptly returned to the hospital resource pool for reallocation in the next cycle.

#### Restock hospital resources

**Fig 4:**
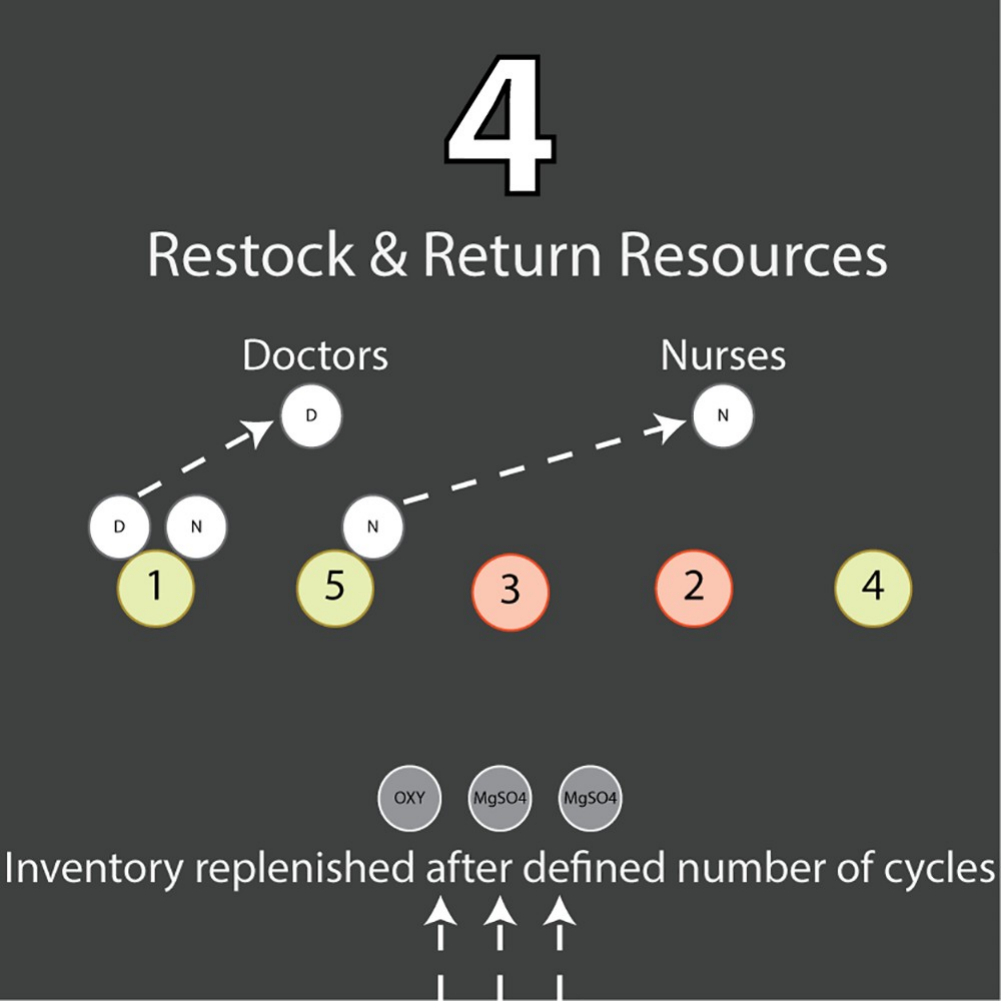
Schematic of the fourth and final step in the algorithm, before the four steps are repeated for the next fifteen minute cycle—medications are restocked if necessary, and any doctors or nurses that were treating patients in the current cycle but are not required for treatment in the next cycle are returned to the staff pool to be re-allocated in the following cycle.

The last phase of the cycle accounts for the hospital restocking its inventory, updates to on-duty staff based on shift changes, and for changing patterns of patient admission at different times of day (morning, evening, and night) (Fig 4). If the model simulates several months at the maternity ward, it must account for new shipments of medication and consumables at defined intervals of time, which can be set by the user or will default to monthly restocking of the hospital inventory.

#### Model outputs

In order to store the multi-dimensional data generated by the model in a flexible and organized format, the model outputs were stored in a JavaScript Object Notation (JSON) file. A user interface for plotting data and exporting the data to other file formats was developed in Java to enable efficient parsing of the JSON file and identification of trends of interest. The number of maternal deaths outputted by the model for each test case was obtained from 50 trials to capture the impact of stochastic variations.

#### Model calibration

The model was calibrated by inputting current medication inventories (as mentioned above in the Methods section), with monthly restocking of oxytocin and hydralazine, and the staff available at the MMH maternity ward. The staff available in the ward and rate of patient admission varies between the morning, evening and night shifts, and this variation was incorporated in the model during calibration (Table 2). All model runs were executed with these parameters, unless otherwise specified.

**Table 2:** Variations in staff capacity and probability of patient admission at Mnazi Mmoja Hospital’s maternity ward over a 24 hour period.

Shift	Number of Nurses	Number of Doctors	Probability of Patient Admission in 15 minutes
Morning	5	3	0.4
Evening	3	2	0.28
Night	3	1	0.625

**Fig 5:**
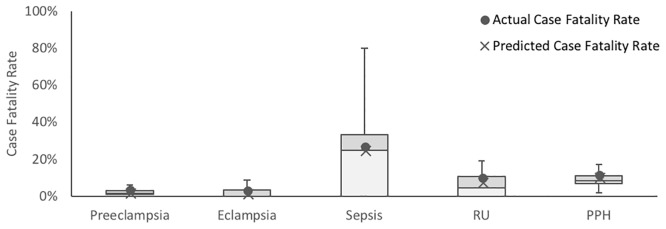
Comparison of case fatality rates for each complication outputted by the calibrated model and case fatality rates recorded by Herklots et al for the five complications incorporated in the model. Box plots represent distribution of data over n = 50 identical simulations. Outliers have been omitted from the box plots for clarity. Predicted case fatality rate is depicted as the mean of each data set, inclusive of outliers.

**Fig 6:**
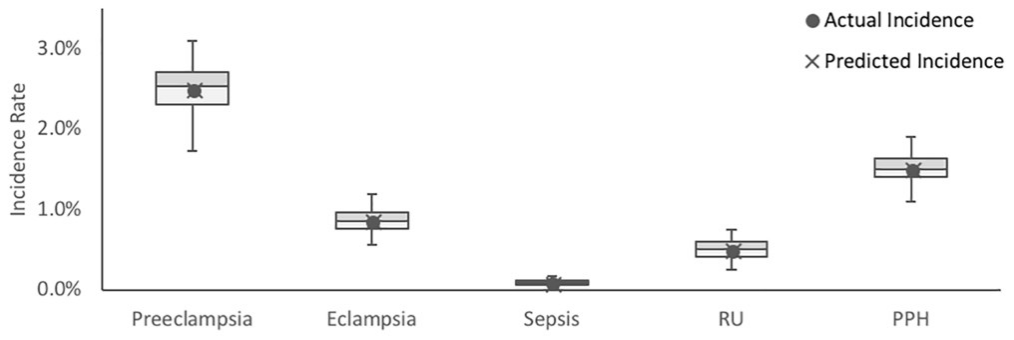
Comparison of complication incidence rates outputted by the calibrated model and complication incidence rates recorded by Herklots et al for the five complications incorporated in the model. Box plots represent distribution of data over n = 50 identical simulations. Outliers have been omitted from the box plots for clarity. Predicted case fatality rate is depicted as the mean of each data set, inclusive of outliers.

With these inputs, deterioration rates for complications were adjusted such that case fatality rates and complication incidence rates outputted by the model over three simulated months matched those obtained from a clinical study conducted by Herklots et al at the MMH maternity ward (Figs 5 and 6) [16].

## Results and discussion

Our model provides a new and distinctive tool that allows hospital administrators and health officials to track the impact of resource limitations on mortality rates in local hospitals. Here, we demonstrate the application of this model on an analysis of the maternity ward at Mnazi Mmoja Hospital in Stone Town, Zanzibar, Tanzania. Unlike population level analyses that review resource uses as a whole, our simulation-based approach enables users to investigate how resource consumption is impacted by individual stochastic events involving patient admission rates and associated complications and severities at admission. Additionally, by tracking and treating individual patients, the model can help to understand the impact of cycles of successful and unsuccessful treatment due to limited resources and identify critical factors that may help prevent these cases from becoming fatalities. Simulations of three-month durations at MMH were conducted to understand the influence of three major factors: medicine restocking schedules, amount of staff on duty, and patient influx.

To verify the model was considering relevant patient loads, we examined the average number of patients admitted to the ward over one month, the average number of patients in the ward per day and the proportion of patients with potentially life-threatening complications. The results showed an average of 1149 patients admitted per month and falling into the range reported by MMH, which varied from 748 patients in December 2016 to 1494 patients in April 2017; however, it is higher than the average of 925 patients per month found by previous studies [16]. The steady-state population of patients in the ward at any given moment was 35 with a standard deviation of 4.7 patients. From monthly records kept at the ward, the average number of patients per day varies between 24.9 patients per day in December 2016 and 49.6 patients per day in April 2017. The 6981 patients recorded in the ward between April 2016 to October 2016 by Herklots et al corresponds to an average of 38.1 patients per day [16]. Based on these findings, the model was found to closely replicate the characteristics of the patient cohort at the MMH maternity ward, and thereby accurately reflect the demand for medication and staff resources seen at MMH.

### Effects of essential medicines availability on maternal mortality

To demonstrate the utility of the model in informing procurement of essential medicines at the maternity ward, the effects of two of the most frequently used medications–oxytocin and hydralazine–were examined. At MMH, all patients in delivery are given prophylactic doses of oxytocin, and additional doses are administered to patients who experience postpartum hemorrhage or are designated as high risk. As an antihypertensive, hydralazine is administered to patients with severe gestational hypertension, severe pre-eclampsia and eclampsia to lower the blood pressure and prevent life-threatening complications, mainly cerebral hemorrhage, as per the standard hospital protocol at MMH. From pharmacy inventory records at MMH between August 2016 and June 2017, there had been three non-consecutive months with a stock out of oxytocin and five non-consecutive months with a stock out of hydralazine. Restocking of both medications was irregular, varying from monthly to every two or three months. Additionally, the amount of medication received was found to be inconsistent between shipments.

As such, to determine the effects of oxytocin and hydralazine availability on maternal mortality, the model was run with varying initial supplies of each medication and compared monthly restocking to no restocking over a 3-month period. When varying oxytocin stocks, the initial hydralazine was set to an 11-pack stock (approximately 2200 mg/mL of hydralazine) and not resupplied during the quarter to inspect the relative impact of the two medications on maternal mortality. Conversely, when varying hydralazine stocks, the initial oxytocin stock was set to 200 packs (approximately 20,000 IU of oxytocin) and was not resupplied during the quarter. In all cases, the model was run with the staffing distribution and patient admission rates detailed in Table 2.

**Fig 7:**
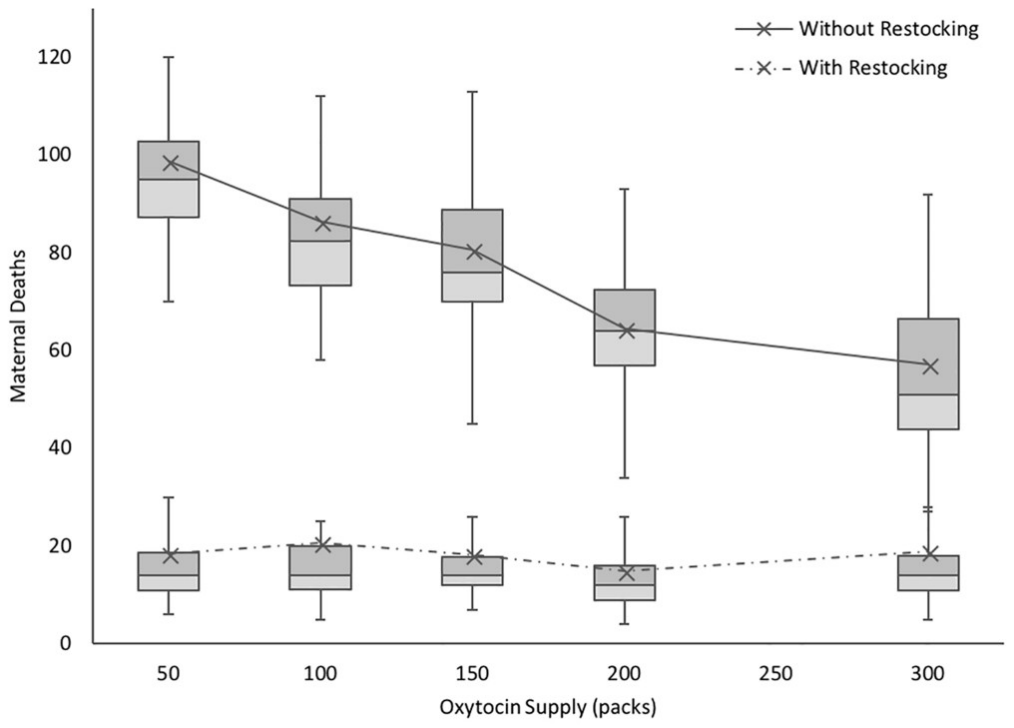
Impact of oxytocin inventory and supply frequency on maternal deaths over a 3 month period. Box plots exclude outliers and whiskers reflect local maxima and minima among n = 50 runs for each data point. Line plots reflect mean values for each data point, including outliers.

For oxytocin, our model shows that monthly restocking results in the greatest improvement on the number of maternal deaths over 3 months (Fig 7). It also indicates that a single large shipment that meets the demand for oxytocin over three months may contribute to worse outcomes than smaller, more frequent shipments, due to stochastic demands for oxytocin supplies. The majority of shipments of oxytocin to MMH tend to be between 150 packs and 200 packs—and as such, missing a shipment is related to significant, detrimental effects on maternal mortality outcomes. The predicted number of maternal deaths increases almost five-fold when the 150-pack stock is not replenished in a 3-month period, underscoring the importance of investing in a steady, reliable supply chain to improve maternal health outcomes.

**Fig 8:**
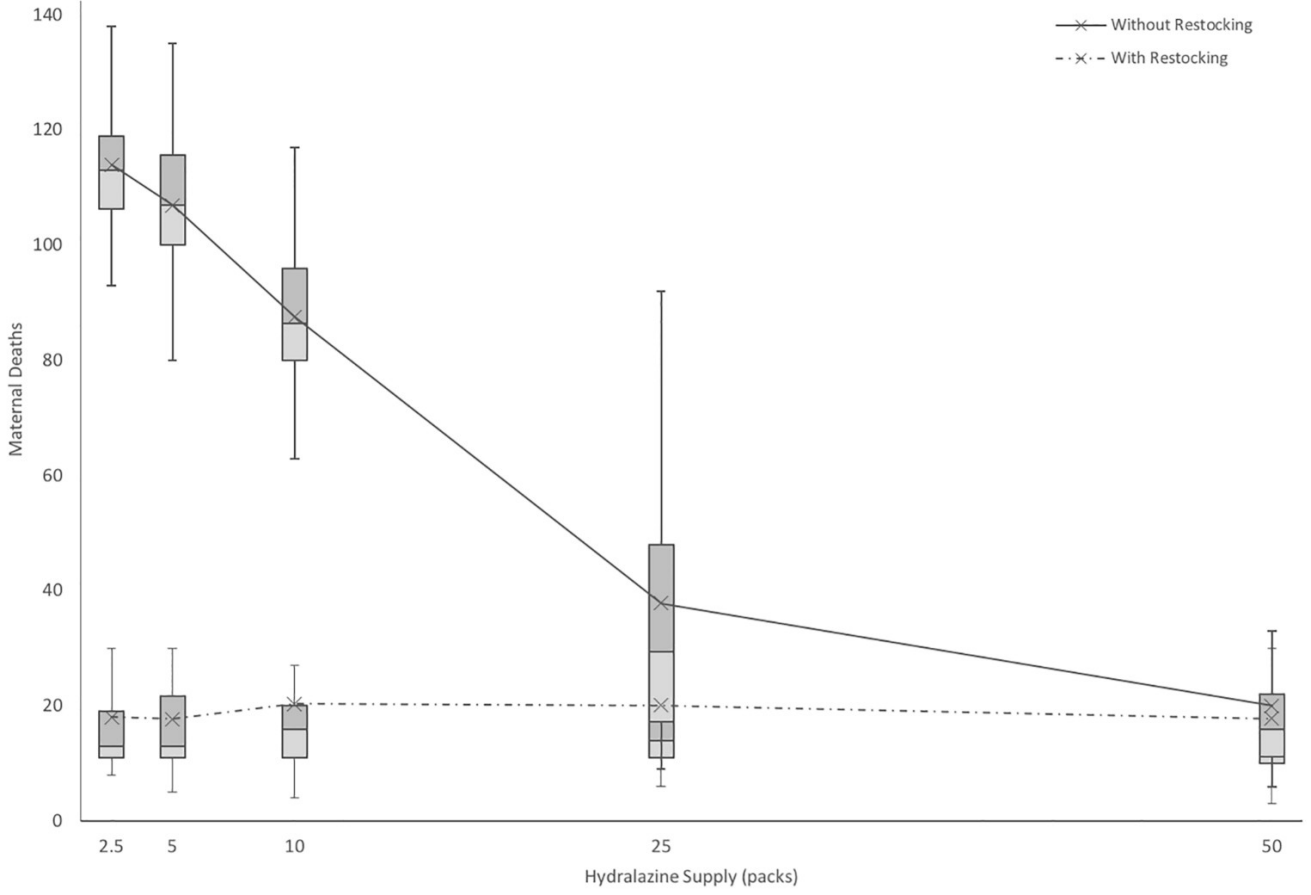
Impact of hydralazine inventory and supply frequency on maternal deaths over a 3-month period. Box plots exclude outliers and whiskers reflect local maxima and minima among n = 50 runs for each data point. Line plots reflect mean values for each data point, including outliers.

Testing for hydralazine shows a similar trend in the necessity for restocking if smaller shipments are supplied, with the number of maternal deaths increasing over four-fold when 10 packs of hydralazine are not replenished over three months (Fig 8). The absolute number of maternal deaths remains high after 25 packs of hydralazine are supplied regardless of restocking, indicating that while hydralazine is crucial for maternal health, other factors, such as staff limitations and oxytocin, contribute more significantly to adverse maternal health outcomes.

### Effects of staff availability on maternal mortality

Adequate monitoring of patients and timely interventions in the case of complications are crucial to preventing maternal deaths, and as such sufficient staffing of the maternity ward plays a key role in decreasing maternal mortality. Surveys of staff scheduling revealed that there are typically five nurses and three doctors on staff at the maternity ward, while at night there are on average two nurses and one doctor. However, additional reports from staff describe extremely limited staff availability—with only one nurse staffing the ward at times or no doctors available in the ward. Given this, we chose to examine the effects of staff capacity on maternal mortality and determine minimum staffing requirements necessary to minimize maternal deaths, keeping in mind the different rates of patient admission during the morning, evening, and night.

**Fig 9:**
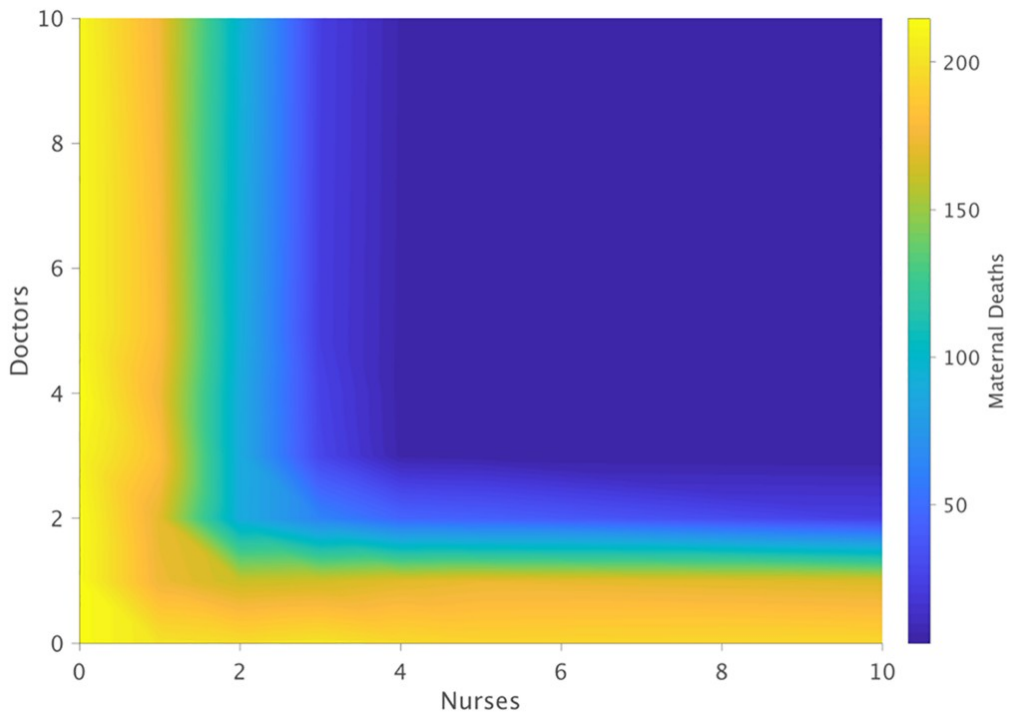
Predicted effect of staffing combinations for nurses and doctors when staffed consistently across morning, evening, and night shifts. Data depicts mean maternal deaths for each staff combination over n = 50 identical simulations of over a simulated timeframe of 3 months with 20,000 IU of oxytocin and 2200 mg/mL of hydralazine being supplied each month.

The number of nurses and doctors available to staff the ward was varied between 0 and 10 and simulations were run over a 3-month period to determine the impact on maternal deaths. Fig 9 summarizes the results and demonstrates that under extreme staff shortages, the number of maternal deaths can reach over 200. Adding additional staff drastically reduces these deaths from over 200 to under 50, however the plot indicates that this can only be achieved if a critical number of both doctors and nurses is achieved. Under the specific morbidities and associated treatment plans considered for this model, the minimum number for both doctors and nurses was found to be 3. Fig 9 shows that when staffed with only 2 doctors, additional nurses have minimal to no effect on the number of deaths. Similarly, when staffed with only 2 nurses, additional doctors also have minimal to no reduction in deaths. Once a minimum of 3 doctors and 3 nurses has been met, we find that increases in staff lead to further decreases in mortality, particularly when adding nurses. This corroborates well with the observations of the hospital staff and their experience and is also consistent with the fact that in this model, nurses are required at a higher frequency for continual patient care than doctors, as defined within the specific treatment plans for each condition. Again, it should be underscored that the critical number of doctors and nurses required to adequately manage maternal mortality are heavily dependent on complication incidence rates, specific treatment plans, and overall patient load. While at MMH our model finds this number to be 3 for both doctors and nurses, the model should be used as a tool to continually evaluate these critical numbers under changing conditions at the hospital or under different conditions at another hospital.

**Fig 10:**
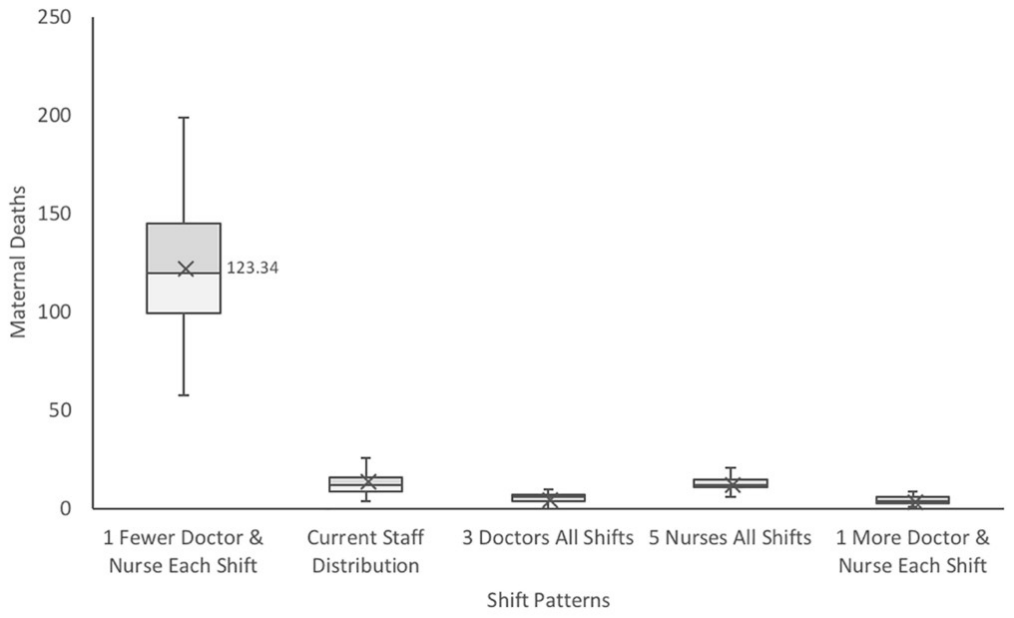
Effect of different staffing distributions during the morning, evening, and night on maternal mortality. Inset depicts zoomed version of shift patterns excluding the 1 fewer doctor and nurse condition. All data sets were found to be statistically significant from one another, except for the current staff distribution compared to the 5 nurses for all shifts, with p < 0.05 using a two-tailed test.

To explore the effects of different staffing schedules on maternal mortality, we varied the number of staff available on each shift from the existing distribution detailed in Table 2. As observed in Fig 10, decreasing the staff capacity on each shift by even one doctor and nurse contributes to an increase in the number of maternal deaths by almost ten-fold, attesting to the importance of ensuring the critical number of staff members are available at all hours. Increasing the number of nurses available during the evening and night shifts at the ward correlates to outcomes comparable to the status quo. However, increasing the number of doctors available on all shifts to the critical number of 3 corresponds a 50% reduction in maternal deaths, indicating a potential insufficiency in the number of doctors available in the evening and at night.

### Effects of patient influx on maternal mortality

As the main referral hospital in Zanzibar, MMH receives a range of patients from self-referrals to patients who are referred from other health facilities. The spectrum of severity ranges from relatively uncomplicated and non-severe cases that ideally would seek care in the secondary or primary health care facilities, to severely complicated cases for which MMH is the designated hospital. The large influx of patients exacerbates delays in receiving care, stock-outs of medical supplies and shortages of staff. We examined the effects of decreasing the rate of patient influx to the maternity ward from 0.4 patients per 15 minutes to assess how this affects mortality outcomes for patients with potentially-life threatening complications. Conversely, the effects of a higher patient influx to MMH’s maternity ward were examined to determine if the ward could respond to higher patient volumes in the future without compromising health outcomes. Currently, approximately 50% of pregnant women in Zanzibar deliver in health facilities [22]. With public health initiatives encouraging women to deliver in health facilities, the health system must have the capacity to respond to the increasing need for care.

**Fig 11:**
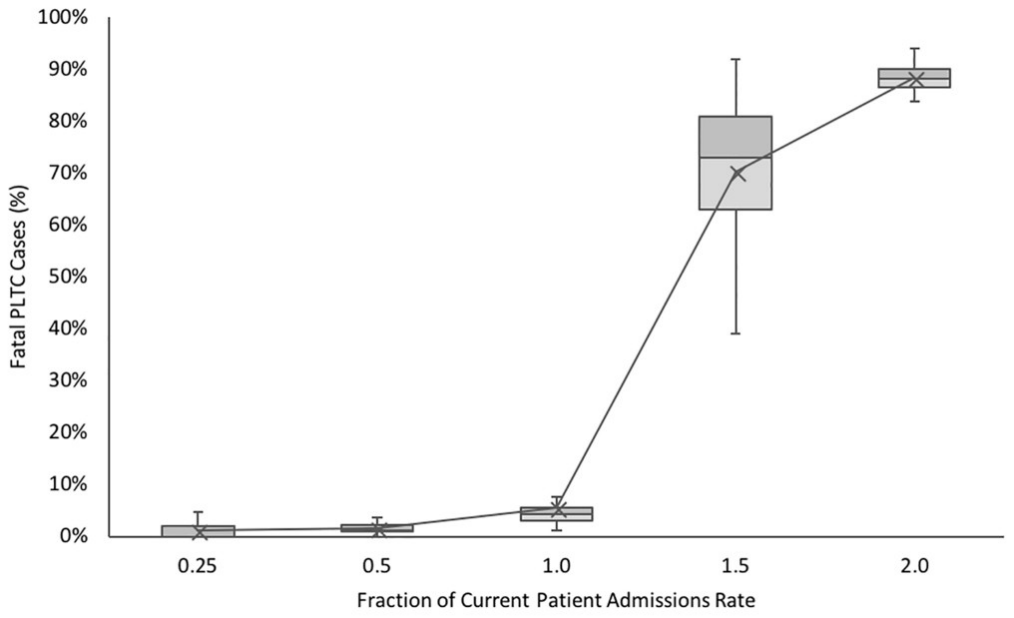
Predicted effect of patient influx rate on mortality rates for patients with complications.

Simulations indicate that a patient admission rate that is half of the current patient admission rates in the morning, evening and night, contributes to a decrease in mortality rate for patients with potentially life-threatening complications from 5.53% to 1.60% (Fig 11). Increasing patient admission rates by a factor of 1.5, however, increases the mortality rate among high-risk patients by over fourteen-fold. This supports a shift to treatment of low-risk patients at secondary health care facilities, freeing up resources in the MMH maternity ward for high-risk patients in order to improve overall maternal health outcomes. It is of key importance that those facilities designated to deal with a potential increased patient load will be fully equipped and staffed to do so.

### Conclusion

The health systems model described here was created for use in the Mnazi Mmoja Hospital maternity ward in Zanzibar, Tanzania. Yet, it provides a template for health systems modeling at other facilities, providing policymakers and hospital administrators with a tool to identify gaps and inefficiencies in maternal care that can be addressed in order to improve maternal mortality. The model has identified the following factors as contributors to a reduction in maternal mortality in this setting: (1) more frequent, monthly shipments of oxytocin and hydralazine to health facilities as opposed to larger, quarterly shipments, (2) an increase in the number of nurses working per shift, assuming the critical number of 3 doctors per shift has been met, and (3) a reduction of patient admissions by directing low-risk cases to deliver at primary and secondary healthcare facilities outside of MMH.

### Assumptions, limitations, and future directions in health-facility modeling

While the model accounts for the staff capacity, medication inventory, treatment plans, and triaging of patients for six noted conditions, collection of additional clinical data likely will extend the utility of the model by accounting for the use of the intensive care unit, blood products, non-pharmaceutical consumables, surgical tools, and medical equipment. The model does not account for patients procuring their own medication and consumables for use in the event of stock outs (though this occurs at MMH), and operates under the ideal case of all medication being procured from Zanzibar’s Central Medical Store and purchased using funds from the government or aid agencies. Temporal changes in staff capacity and physical separation of antenatal wards, labor rooms, and postnatal wards were not included in this iteration of the model. Building on this framework to include these factors could extend the model for further use as a tool to optimize logistics and operations in the maternity department.

Age and obstetric history of the simulated patients do not affect their initial probability of mortality in this iteration of the model, as further data must be collected to assess the relationships between age, prior complications in pregnancy, and mortality in order to be incorporated in the model. Accuracy of diagnosis was not incorporated into this version of the model, as it is assumed that if a patient has a complication that is what they are diagnosed with and treated for. Patients in the model can only have one of the five complications, if any, and cannot develop new complications as they progress through the simulation. Additionally, because there is insufficient data on how to weigh the impact of each resource prescribed by the treatment, all resources necessary for treatment must be available in the simulated ward inventory and staff pool for a patient in the model to be treated.

Cesarean sections, hysterectomies, laparotomies, salpingectomies, and oophorectomies performed at the maternity ward are indirectly included in treatment plans but only specify the staff resources needed and the treatment efficacy; the average length of time of the surgery, the need for and availability of surgical tools and an operating theatre were excluded from this iteration of the model. Further, the potential for a patient’s condition to worsen due to complications arising from surgery was not included in the model due to a limited number of post-surgery complications becoming fatal. Abortions and anemia were not explicitly incorporated in the model as complications, though both occur in a substantial proportion of patients. Anemia has a relatively low mortality rate relative to other complications included in the model, and there was not adequate information on patient abortions in the medical records.

Despite these limitations, we note that the model was able to capture the current trends and provide specific predictions that are relevant for understanding the complex operation of the maternity ward. We also note that by comparing the resource allocation and usage of human, pharmaceutical and capital resources, we are able to analyze the varying burdens on resources. Our model is unique from other generic tools for improving efficiency since it incorporates local challenges, patient trajectories and observations of staff. While the model is based on patient outcomes at MMH in Zanzibar, we note that the framework is modular and capable of adaptation to other wards and hospitals in low and middle-income countries using facility-specific inputs.

## Supporting information

S1 Table Deidentified patient data collected from the maternity ward in Mnazi Mmoja Hospital, Stone Town, Zanzibar, Tanzania. (XLSX)

S2 Table Treatment trajectories for six maternal health conditions, ranging in severity, as described by clinicians in Mnazi Mmoja Hospital, Stone Town, Zanzibar, Tanzania. (XLSX)

S1 Text Github URL location of the script for the model system. (DOCX)
