# Supplementary material for: Predicting resource-dependent maternal health outcomes at a referral hospital in Zanzibar using patient trajectories and mathematical modeling
Source: PLoS One. 2019 Mar 5;14(3):e0212753. doi: 10.1371/journal.pone.0212753 (PMC6400335; doi:10.1371/journal.pone.0212753)
Supplement: S1 Text — (DOCX) [file pone.0212753.s003.docx]

Link to code for the model:

<https://github.com/randybme/MMHModel>
